# The Red Cross

**Published:** 1882-08-20

**Authors:** 


### EDITORIALS AND MISCELLANEOUS.

☞ THE RED CROSS on your Journal shows that you are in arrears for subscription.

